# Can Professionalization Alleviate Job Burnout in Construction Workers in China? A Multivariable Mediating Model

**DOI:** 10.3390/ijerph192113879

**Published:** 2022-10-25

**Authors:** Guodong Ni, Xinyue Miao, Li Li, Huaikun Li, Shaobo Wang, Miaomiao Niu

**Affiliations:** 1 School of Mechanics & Civil Engineering, China University of Mining and Technology, Xuzhou 221116, China; 2 Research Center for Digitalized Construction and Knowledge Engineering, China University of Mining and Technology, Xuzhou 221116, China

**Keywords:** job burnout, construction worker, professionalization level, workload, job insecurity, work–family conflict, structural equation modeling

## Abstract

Burnout is at all-time highs across modern professions. As a typical labor-intensive industry, the high-pressure and task-driven nature of the construction industry makes construction workers more prone to burnout. It is still unclear whether increasing the professionalization level can lessen the many harmful consequences of job burnout on construction workers’ employment. Therefore, this study examined the influencing mechanism of professionalization on job burnout in the construction industry. First, a theoretical model based on the conservation of resources (COR) theory was developed with workload, job insecurity, and work–family conflict as moderating variables. A reliable sample of 441 Chinese construction workers were then recruited in the investigation. The data analysis was supported by confirmatory factor analysis (CFA) and structural equation modeling (SEM). The results indicated that: (i) an increase in the professionalization level could be directly effective in alleviating job burnout among construction workers; (ii) workload and work–family conflict could play an independent and continuous mediating role between professionalization and job burnout; and (iii) while job insecurity caused by a low professionalization did not have a direct impact on job burnout, it could have an indirect impact on job burnout through workload and work–family conflict, respectively. This study enriches the literature on job burnout among construction workers, as well as provides a theoretical basis and practical management guidance for Chinese construction companies to alleviate job burnout in workers from a professionalization standpoint.

## 1. Introduction

Due to the high levels of stress and its task-driven environment, the construction industry is considered as one of the most hazardous and unhealthy industries [1,2]. Construction projects are often large-scale, complex, integrated, and involve multiple stakeholders. Practitioners are always exposed to a high level of risk and are required to cope with a lot of complex tasks in a limited time [3,4]. In addition, there are always complicated work relationships under a demanding and stressful work environment during the whole project life cycle [5]. Prolonged work under great stress can continuously and rapidly deplete the physical and psychological resources of workers, harm their health and potentially lead to job burnout [6].

Job burnout has been a significant problem for the workforces of modern society [7]. Excessive stress, insecurity, undervaluation, and alienation in the workplace can all trigger psychological problems among workers for whom job burnout may develop [1,7]. In the construction sector, there is also a need to be careful about job burnout. Previous research has shown that job burnout, an important attrition factor in the Job Demand-Resource (JD-R) model, not only has a negative impact on practitioners’ health and well-being but can also lead to unsafe behaviors and affect project performance [8]. Therefore, it is critical to address the burnout that Chinese construction professionals are experiencing.

Around 220 million people worldwide are employed in the construction industry, which is labor-intensive, with non-nationals and migrant workers making up a sizeable part of this workforce [9]. This tendency is particularly noticeable in China. In the rapid process of industrialization and urbanization, migrant workers have emerged as a unique product of China’s dualistic economic system [10]. They usually have a rural household registration, but work in non-agricultural industries in the city, with the ambiguous status of “half-agricultural and half-worker” [11]. Due to the low entry barriers and few technical requirements, the construction industry has attracted a huge amount of rural labor, making it a primary choice of profession for less-skilled migrant workers [10]. According to the ‘2021 national migrant workers monitoring survey report’ released by China’s National Bureau of Statistics, the construction industry accounts for 19.0% of migrant workers’ employment, second only to the manufacturing industry, with predominating physical labor works. Migrant laborers are the front-line workers and direct finishers of building products in the construction industry. They have greatly assisted China’s urbanization and social development. However, compared to the industrial workers in developed countries with high technical quality, stable positions, high social status, and legal labor security rights, migrant workers in China’s construction industry also have many problems, which hinders the sustainable development of the construction industry [12]. On an individual level, the common stereotypes of migrant workers include being uneducated and lacking vocational skills; on an organizational level, the unprotected working environment, high mobility of personnel, and lack of safety training all pose great challenges for organization management, which may even affect construction safety [11,13]. In addition, migrant workers in the construction industry suffer from low social status, difficulties in protecting their legal rights and interests, and often are without a sense of belonging, all of which have a negative impact on the healthy development of the industry [14]. Therefore, it is urgent to promote migrant workers for professional transformation in the construction industry of China.

Job burnout is often caused by excessive job demands [15]. In contrast, according to the professionalization and JD-R theory, an increased professionalization level can create quality employment and an improvement in the job conditions for construction workers, as evidenced by a reduction in job demand and an increase in job resources. It is thus reasonably presumed that a higher level of professionalization might better balance the relationship between job demands and resources and thus alleviate workers’ job burnout. Numerous factors have been linked to job burnout during the past few decades. The key contributors to burnout include perceived workload, role ambiguity, role conflict, physical discomfort, emotional demands, job instability, and work–family conflict as job demands [5,16,17]. In addition, the factors influencing job burnout are not completely isolated from each other but are rather interactive and reciprocally embedded [5].

Many academics have studied approaches for reducing construction workers’ job burnout. Most existing studies on job burnout have paid attention to exploring the causality of job burnout, and much research on antecedent variables has examined the effect of one or a few variables on job burnout based on the conservation of resources (COR) theory. However, few studies have analyzed whether the transition of migrant workers to industrial workers in the construction sector has improved their job burnout in a systematic way. Moreover, few studies have conducted in-depth research on the interactions between the main factors that contribute to job burnout. To address the research gaps, this study developed a theoretical model based on the COR theory to explore the interactions among professionalization, workload, work–family conflict, and job burnout from the perspective of professionalization. Structural equation modeling (SEM) was used for empirical data analysis.

The objectives of this study were as follows: (i) to analyze the association between professionalization and job burnout, and (ii) to analyze the interactions between workload, job insecurity, and work–family conflict and how they impact the relationship between professionalization and job burnout.

## 2. Theoretical Background

The COR theory has gained popularity in the realm of organizational psychology and organizational behavior over the past 30 years, it being one of the most cited [18]. According to this theory, people experience stress as a result of resource depletion without replenishment [19]. To prevent resource loss or even depletion, recover from losses, and acquire new resources, people need to make investments [18]. The COR theory is a general stress theory often used in explaining job burnout [20]. According to the COR theory, poorly organized work and high work demands can gradually deplete personal physical and mental resources. Higher degrees of burnout may come from employees having a harder time dealing with their job demands, including workload and emotional pressures, due to chronic physical and psychological issues [19]. This study examined whether it is possible to reduce the resource consumption of construction workers and alleviate their workload, job insecurity, and work–family conflict, and thus reduce their job burnout by improving their professionalization level.

### 2.1. Job Burnout

Job burnout is considered to be an occupational psychological syndrome [21]. Maslach and Jackson [22] conducted research on this topic among individuals who engage in “people-work” of some kind and proposed three dimensions of it, including emotional exhaustion, depersonalization, and personal accomplishment, which was supported by the research of Yang et al. [23]. In recent years, many studies have been undertaken to analyze the antecedent variables of job burnout based on Maslach’s theory. Yue et al. [24] suggested that job burnout stems from the dual pressures of task goals and communication/coordination. In contrast, Tong et al. [25] proposed that it is a psychological syndrome experienced by employees after prolonged exposure to negative emotional and interpersonal stressors at work.

In the construction industry, researchers have noticed that job burnout not only has a serious impact on the everyday life and work performance of workers, but also on the organizations in which they work [15,24]. Hence, many studies have been conducted on this topic. Particularly, construction workers are more likely to suffer from job burnout as a result of high workload, long working hours, and high safety risks [24]. The constraints of time pressure, coupled with limited resources, role conflict, and role ambiguity, make construction professionals have a high level of job burnout [26].

### 2.2. Professionalization

More and more industries are becoming professionalized following the continuous refinement of labor division, and professionalization has become an indicator of the industry’s maturity [27]. The current research on professionalization mainly focuses on three perspectives: characteristic factors, process theory, and rights theory. Most researchers generally agree that the process theory perspective most closely resembles the concept of professionalization, which is seen as the evolution of a profession from a completely unstructured job to a fully developed one [28,29]. Furthermore, according to Wilensky [28], professions generally undergo the following stages: creating full-time jobs, establishing training schools, founding professional associations, developing relevant laws and rules, etc.

Migrant workers are a special social phenomenon during the period of social transformation in China, so achieving their transformation into industrial workers has become an inevitable requirement and a strategic task to promote the industry and its development [30]. In line with previous research is defining the dynamic process of professionalization in relation to the intellectual skills, professional, ethics and social status of the employee [31]. Furthermore, some psychological driving factors such as perceived behavioral control, subjective norm, and behavioral attitude have been shown to influence the development of construction workers’ professionalization [11]. The professionalization of construction workers is therefore a process of improving job skills, developing vocational qualities, observing professional norms, and acquiring appropriate status and security in the modern construction sector [28,32].

### 2.3. Workload

Some researchers have focused on the concept of workload as the number of tasks per unit of time [33]. Jones & James were early proponents of the idea of role overload, which they defined as the degree to which performance is impacted by inadequate resources, training, and time to perform one’s role [34]. The workload can refer to different yet related constructs such as job demands and job overload. The two interrelated structures of job demand and job overload are related to workload [33]. MacDonald [35] categorized the demand factors impacting workload as physical, sensory, central processing, psychomotor, and affective. Jobs become stressful when people are required to carry out more work than they have resources for, which will further negatively impact burnout, anxiety, and depressive symptoms. This is compatible with the COR theory’s perspective [36,37]. Moreover, current studies generally believe that workload will consume individual physiological and psychological resources [38]. As for construction workers, workload imposes both a physiological and psychological drain on them due to the high-intensity, dangerous, and complex work they carry out for an extended time, which may further result in exhaustion [39]. In a word, the workload depends on the job demand and ability of workers, and when the work is too heavy or the difficulty of the task exceeds workers’ ability, it will have both psychological and physiological impacts on them.

### 2.4. Job Insecurity

Job insecurity was earlier defined as an individual’s perceived inability to preserve desired continuity in a threatening employment position [40]. Similarly, Zhang [41] and Yi et al. [42] argued that job insecurity is a lack of security in terms of job continuity or aspects of work. More simply, insecure workers are aware of the possibility of losing their jobs [43]. Indeed, for many people, sustained employment provides a source of income, friendship, a sense of belonging and social status, and access to the above material or psychological resources can be threatened when workers anticipate that their employment may not be sustainable [40,44]. Issues such as temporary forms of employment and low contracting rates make construction workers highly fluid and unprotected. Hence, construction workers tend to be more insecure about their jobs. Job insecurity in this study therefore can be qualified as a concern of job instability and the absence of labor rights protection [11]. This can have a serious negative impact on their psychology and behavior. Previous research has confirmed that job insecurity can contribute to more job withdrawal and poorer task performance among construction workers, which in turn reduces job performance [43,45].

### 2.5. Work–Family Conflict

Work–family conflict is a product of economic development and urbanization in the post-industrial era. This concept was initially proposed by Kahn et al. [46], while Greenhaus and Beutell’s notion of work–family conflict [47], based on role theory and the scarcity hypothesis, is widely accepted as a role conflict arising from role incompatibility between work and family. In other words, work–family conflict refers to the fact that the pressure arising from an individual’s involvement in work (family) activities makes it difficult to fulfil the other role. The sources of work–family conflict include strain-based conflict, time-based conflict, and behavior-based conflict [47].

As a labor-intensive industry, the construction sector absorbs a large amount of surplus rural labor, and the collective mindset drives workers to sacrifice more time to work for a better household in the long run [48]. Furthermore, workload, task-related stress, and flexibility in scheduling all have an impact on work–family conflict [49]. Based on the aforementioned findings, this study viewed work–family conflict as a role conflict caused by employment characteristics and industry features that make it impossible for construction workers to combine work and family life, mainly in terms of the effects of work on the family. In addition, work–family conflict is also regarded as a two-way concept, with both directions related but not identical (i.e., family interference with work is distinct from work interference with family) [50].

## 3. Research Hypothesis and Theoretical Model

### 3.1. Professionalization and Job Burnout

Job demands and job resources are two categories of job characteristics [51]. The former refers to job demands that continuously deplete personal resources or energy; the latter, to resources made available at work to accomplish work goals, lessen job demands and the physiological and psychological costs associated with them, or promote personal growth, learning, and development [52]. As one of the most influential theories on job stress and burnout, the COR theory confirms that job demand is a significant prelude to job burnout by interpreting the causes and processes of job burnout from the perspective of demand and resource acquisition [53,54]. An increased professionalization level can lead to high-quality employment for construction workers, including a reduction in the demand for work, which helps to ease the attrition process and thus effectively prevents workers from developing burnout. Based on the preceding arguments and research evidence, the following hypothesis is formulated:

**Hypothesis** **1** **(H1).** *Professionalization has a significant negative effect on the job burnout of construction workers.*

### 3.2. Professionalization, Workload, and Job Burnout

Stimuli such as the environment and events at work may cause people to develop certain psychological reflections and might have a corresponding effect on their attitudes and behavior [55]. As a labor-intensive industry, the construction industry is characterized by long working hours and high labor intensity. Long-term high-intensity workloads are highly likely to lead to job burnout among construction workers and may further influence their unsafe behavior. Li et al. [56] demonstrated a positive correlation between workload and emotional exhaustion in a study of the link between job demands and resources with safety outcomes (i.e., workplace injuries and near-misses) among crude oil production workers; a similar view is held by Ziaei et al. [57]. The technological upgrades and changes in the mode of production caused by the modernization of construction can reduce the labor intensity and difficulty of construction workers, which, as one of the necessary conditions for the professionalization of the workers, can alleviate their workload to some degree and reduce physical and psychological resource consumption, further acting as a disincentive to job burnout. Therefore, the hypothesis is proposed as follows:

**Hypothesis** **2** **(H2).** *Workload mediates the effect of professionalization on the job burnout of construction workers.*

### 3.3. Professionalization, Job Insecurity, and Job Burnout

Construction workers’ job insecurity mainly derives from the unregulated employment forms and unstable employment relationships in the construction industry, and the prevalence of low socio-economic and educational levels among construction workers can increase their likelihood of experiencing higher levels of job insecurity [58,59]. It is logical to assume, as such, that raising the professionalization level could go some way towards easing the above problems to make workers feel less insecure at work. In addition, many studies have been conducted to support the positive association between job insecurity and job burnout, mainly using the COR theory [42]. When employees’ psychological resources are excessively depleted, it can create extreme stress and consequently negative attitudes towards work and the organization, leading to emotional exhaustion [60]. It is therefore reasonable to hypothesize that workers’ job insecurity is directly influenced by their professionalization degree and may further trigger job burnout. The following hypothesis is put out in light of the analysis above:

**Hypothesis** **3** **(H3).** *Job insecurity mediates the effect of professionalization on the job burnout of construction workers.*

### 3.4. Professionalization, Work–Family Conflict, and Job Burnout

Temporality, complexity, mobility, and the demanding working environment of construction projects have a high potential to negatively impact the family lives of those working in the construction industry [61,62]. According to inter-role conflict theory, when individuals have multiple roles to juggle, potential conflicts between roles can arise if limited resources are not allocated appropriately to the different roles [46]. Thus, work–family conflict arises when the job demands of a construction professional conflict with the fulfilment of his or her family responsibilities [63]. They are prone to experience major physiological and psychological health issues due to the stress associated with work–family conflict, such as dissatisfaction, depression, anxiety, and physical stress [64]. These unfavorable feelings can constantly drain the resources of construction professionals and trigger job burnout [65]. In other words, burnout and work–family conflict are positively correlated, and an increase in professionalization level can reduce workers’ consumption and commitment at work and alleviate the conflict and contradiction between work and family, which in turn reduces job burnout. Therefore, the hypothesis is proposed as follows:

**Hypothesis** **4** **(H4).** *Work–family conflict mediates the effect of professional level on the job burnout of construction workers.*

### 3.5. Professionalization, Workload, Work–Family Conflict, and Job Burnout

Workload, as a work demand, increases the consumption of resources in the work sphere, and the finite nature of resources leads to fewer resources being available to meet household needs [66,67]. When resources are insufficient to simultaneously cover the needs of both roles, conflicts between the two domains may occur [66]. Previous research has confirmed that work overload has the most robust relationship with work–family conflict, a finding that is consistent with the COR theory [68]. Furthermore, in conjunction with the elaborations and findings in 3.2 and 3.4, it can be logically deduced that professionalization can influence job burnout through workload and work–family conflict. For construction workers, increased professionalization can reduce their workload, allowing them to devote more resources to their families. When the conflict between work and family is eased, workers’ job burnout can be reduced. Therefore, the hypothesis is proposed as follows:

**Hypothesis** **5** **(H5).** *Workload and work–family conflict play a serial mediating role between professionalization and the job burnout of construction workers*.

### 3.6. Professionalization, Job Insecurity, Work–Family Conflict, and Job Burnout

Construction employees may experience job insecurity caused by hazardous and precarious employment and working conditions [69]. Additionally, factors such as socioeconomic situation, form of employment, and social support all directly impact job insecurity [70,71]. Job insecurity can increase stress and worry on the family sphere, which can exacerbate the effects of work–family conflict [72]. According to 3.4, work–family conflict may exacerbate workers’ job burnout. Hence, it is reasonably presumed that job insecurity and work–family conflict mediate the relationship between professionalization and job burnout in a continuum, although no previous studies have directly demonstrated this. Logically, an increase in professionalization level can improve the employment environment, working environment, and socioeconomic situation of construction workers to some extent. This helps to alleviate the job insecurity of workers, so that they can devote themselves to their family lives in a more stable and positive mood, avoiding conflicts between work and family effectively to prevent job burnout. Therefore, the hypothesis is proposed as follows:

**Hypothesis** **6** **(H6).** *Job insecurity and work–family conflict play a serial mediating role between professionalization and the job burnout of construction workers*.

### 3.7. Professionalization, Workload, Job Insecurity, and Job Burnout

As discussed above, job insecurity is widespread among the construction worker community. A proportion of workers who perceive job insecurity may allay their job concerns by working harder to prove their value in the organization to their managers [73]. Some studies have suggested that workers with higher levels of job insecurity spend more time at work [74]. In addition, workload, as one of the job demands, can exacerbate the effects of job insecurity on job exhaustion [75]. Therefore, it can be argued that professionalization can have an impact on construction workers’ job burnout through the continuous effect of job insecurity and workload. In light of the analysis above, the following hypothesis is proposed:

**Hypothesis** **7** **(H7).** *Job insecurity and workload play a serial mediating role between professional level and the job burnout of construction workers.*

**Figure 1:**
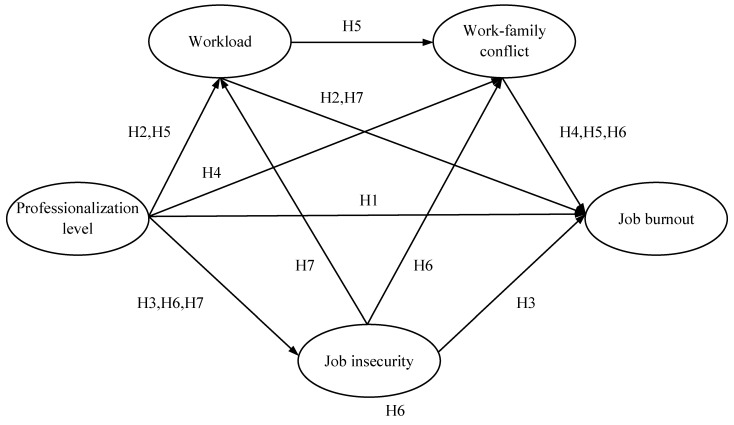
Theoretical model.

According to the hypotheses put forward, the theoretical model of this paper is illustrated in Figure 1.

## 4. Methodology

### 4.1. Measurement Scales

The scales used in this study were adapted from the existing literature to ensure their scientific validity and reliability. Based on the structure and attitude attributes proposed by Wilensky [28] and Hall [76], researchers have mostly divided professionalization into two dimensions (i.e., personal micro level and industry macro level). Therefore, by drawing on and summarizing the existing literature, the scale of professionalization was measured from personal attributes and job characteristics with eleven items. The measurement scales used for workload were mainly developed by Geurts et al. [77], Feng and Ren [38]. The content was adapted to fit the construction industry, resulting in four items. Job insecurity was primarily referenced and modified from Chih’s measurement scale [43], which includes four question items. Combining the characteristics of the construction workers in China, a three-item scale for work–family conflict was adapted from An et al. [78]. According to Wu et al. [24], the measurement scale of job burnout was drawn with three dimensions, including emotional exhaustion, cynicism, and low professional efficacy and consisted of nine items. Because the questionnaire was initially written in English, the authors applied the back-translation method and translated the original instrument into a Chinese version following the Chinese context and asked two professors to examine the face validity of the measurement items before making a basic modification. Then, the authors conducted a pilot test by selecting several construction workers in China to take the survey to examine whether respondents understood the questions in terms of wording and meaning. Following the comments on and results of the preliminary data analysis of the pilot questionnaire, the survey questions were revised. The final Chinese questionnaire for data collecting was created following the pilot and modification. A 5-point Likert scale was used to measure each item on the scale (the score of 1 to 5 means from strongly disagree to strongly agree). The details of the measurement scale can be seen in Appendix A.

### 4.2. Data Collection

Surveys are one of the commonly used methods for performing perception studies [79]. A structured questionnaire was adopted to collect data and test the hypotheses. The survey questionnaire was divided into two sections to fulfil the purpose of the research. The first section was a series of 10 questions about the worker’s sociodemographic information, such as gender, age, marital status, educational degree, location of work, type of work, trade, seniority, working hours in a day, and average monthly income. The second part aimed to explore the relationship between the professionalization and job burnout, containing a total of 31 question items. A combination of online and offline methods was utilized to develop, disseminate, and collect the data. The online research was primarily based on the Questionnaire Star Platform plus WeChat. Meanwhile, offline research was conducted with the assistance of managers on construction sites, who distributed paper questionnaires among workers and guided them to fill them out.

The participation pool included construction workers located across China. The background, purpose, and significance of this study were prominently explained to the interviewees at the very beginning of the formal questionnaire. The interviewees completed the questionnaire voluntarily and knowingly. A total of 1114 questionnaires were collected. Excluding those that did not meet the requirements (such as not having all options filled, answering in a significantly short time, or evaluating each item uniformly or with a clear regularity), 441 valid questionnaires were finally obtained, meeting the sample size that was required.

From Table 1, most of the responding workers were mainly from projects around Jiangsu, Shandong, Sichuan, Hebei, and Henan Province, and they were primarily involved in construction projects (48.3%) and municipal projects (18.4%). The majority of the respondents were men (88.9%), which was in line with the actual situation in the construction industry. The respondents were predominantly carpenters, steelworkers, concrete workers, electricians, and handymen. In terms of educational level, the percentage of workers with education at high school and above reached 57.6%, a reflection of the fact that workers now have a better educational background. Additionally, most respondents had been working for less than a decade in this field, with a concentration of 8 to 10 h per day and an average monthly income of over CYN 5000.

### 4.3. Data Analysis

As there are multiple dimensions of professionalization and job burnout with complex relationships between many of variables, SEM was used in this study to evaluate the hypotheses stated in 3.1. The SEM study was conducted in two main steps according to Anderson and Gerbing [80]: firstly, the design scale was validated by confirmatory factor analysis (CFA), including structural validity, convergent validity, discriminant validity, and internal reliability; after these, the hypotheses formulated in the model were further tested and a thorough path analysis was conducted by AMOS. All the above steps were completed using SPSS 25.0 (IBM SPSS Inc., Chicago, IL, USA) and AMOS 21.0 (IBM SPSS Inc., Chicago, IL, USA).

## 5. Results

### 5.1. Common Method Variance

To avoid common method bias, controlled procedures such as anonymous questionnaires were utilized in the data collection process. Meanwhile, in the process of data analysis, Harman’s single-factor test was used to screen for common method bias [81]. Exploratory factor analysis was applied to all the main variables to examine whether there was a factor that could reveal all the potential variables and the percentage of explained variance where the first factor was located. The results showed the presence of multiple factor structures, while the percentage of the explained variance for the first factor was 44.988% < 50%, indicating that the common method bias problem in this study was not serious [81].

### 5.2. Confirmatory Factor Analysis (CFA)

Confirmatory factor analysis (CFA) is a statistical method used in behavioral sciences to verify whether the data obtained through the questionnaires matches the hypothesis model or not, as well as to identify problematic items on the scale [82]. In accordance with Hair et al. [82,83], the model in this study was evaluated using the fit indicators listed below:

- Absolute fit measures, including observed normed χ^2^ (χ^2^/df), root-mean square residual (RMR), goodness-of-fit index (GFI), and root-mean square error of approximation (RMSEA);
- Incremental fit measures, including normed fit index (NFI), incremental fit index (IFI), the Tacker–Lewis index (TLI) or non-normed fit index (NNFI), adjusted goodness-of-fit index (AGFI), and comparative fit index (CFI);
- Parsimonious fit measures, including parsimony goodness-of-fit index (PGFI), parsimony normed fit index (PNFI) and parsimony comparative fit index (PCFI).

The measurement model fit assessment results and the recommended values for each indicator are shown in Table 2. The results showed that all the indicators met ideal levels [84,85,86], meaning that the model was a good explanation of the data collected.

According to Mueller and Hancock, convergent validity refers to the degree to which two measures of constructs are theoretically related and is always verified by standardized factor loadings (FL > 0.5), construct reliability (CR > 0.7), and average variance extracted (AVE > 0.5) [4,86,87,88]. In addition, the internal reliability of factor items can be verified by further calculating the coefficients of Cronbach’s alpha, which is considered acceptable when the values are higher than 0.7 [89].

The results of the tests of convergent validity and internal reliability are displayed in Table 3. The FL of each item was greater than 0.5, and the CR and AVE of all the constructs were higher than 0.8 and 0.5, which satisfied the test criteria, indicating that the convergent validity was relatively satisfactory. Furthermore, the coefficients of Cronbach’s alpha for all items ranged from 0.808 to 0.971, which exceeded 0.7, implying that the internal reliability was acceptable. Therefore, the results indicated that the model adequately satisfied the convergent validity and reliability criteria.

Following on the results of the convergent validity, the discriminant validity was examined. It represents the degree of difference between two similar terms and is commonly checked by comparing the variables’ AVE square root with the correlation coefficients between the same construct and any other construct. If the former is greater than the latter, then it is considered to have good discriminant validity [90].

Table 4 shows the means, standard deviations (SDs), and correlation coefficients of the variables. The results demonstrated that most diagonal elements were basically higher than their respective off-diagonal elements. Moreover, all *p* values were less than 0.01, indicating significant correlation between the variables. Thus, the measuring model possessed discriminant validity. However, Table 4 also shows that the correlation coefficients between emotional exhaustion and cynicism as well that between cynicism and low professional efficacy were 0.802 and 0.768, respectively, which were slightly above 0.75, so a covariance diagnosis was carried out using SPSS 25.0. The results indicated that the maximum value of the variance inflation factor (VIF) was 4.177, which was significantly less than the recommended cutoff value of 10, suggesting that multicollinearity did not exist between the measured variables.

In summary, the adequate model fit, along with the good convergent validity, discriminant validity, and internal reliability of the model were strong evidence that the measurement model was appropriate for SEM.

### 5.3. SEM Analysis and Hypothesis Testing

The SEM consisted of two components: a measurement model and a structural model. The CFA described earlier provided the measurement model, which primarily tested the relationships between the latent variables and their indicators, while the structural model was the path model linking the independent and dependent variables. In this study, AMOS 21.0 was used to test the research hypotheses and conduct the pathway analysis.

**Figure 2:**
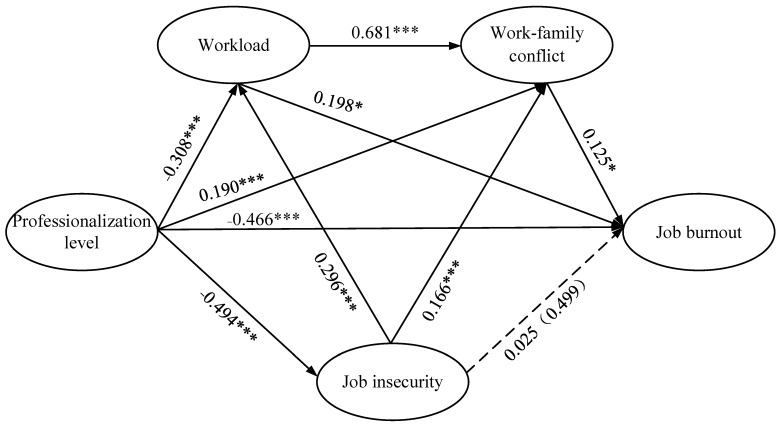
Research model and results of the hypothesis test. Note: the values on the lines are the path coefficients. The value in brackets is *p*. The solid lines and dashed lines indicate passed paths and rejected paths, respectively. ***, *p* < 0.001; *, *p* < 0.05.

According to Figure 2 and Table 5, 6 hypotheses were supported. The effects of professionalization on job burnout (professionalization→job burnout, −0.466, *p* < 0.001), professionalization on workload (professionalization→workload, −0.308, *p* < 0.001), professionalization on job insecurity (professionalization→job insecurity, −0.494, *p* < 0.001), professionalization on work–family conflict (professionalization→work–family conflict, −0.190, *p* < 0.001), workload on job burnout (workload→job burnout, 0.198, *p* < 0.05), work–family conflict on job burnout (work–family conflict→job burnout, 0.125, *p* < 0.05), workload on work–family conflict (workload→work–family conflict, 0.681, *p* < 0.001), job insecurity on work–family conflict (job insecurity→work–family conflict, 0.166, *p* < 0.001) and job insecurity on workload (job insecurity→workload, 0.296, *p* < 0.001) were all significant. Therefore, H1, H2, H4, H5, H6, and H7 were supported and passed the test, while the effects of job insecurity on job burnout (job insecurity→job burnout, −0.025, *p* = 0.499) was not significant, and hence H3 was rejected and could not pass the test.

To further analyze the mediating role played by workload, job insecurity, and work–family conflict between professionalization and job burnout, multiple linear regression analysis in SPSS 25.0 was used for the validation and the results are shown in Table 6 and Table 7. Job burnout and professionalization had a substantial inverse relationship, with the direct effect being −0.434 (*p* < 0.001). When workload acted as the single mediating variable, the lower and upper BC confidence intervals for the specific indirect effect of professionalization on job burnout through workload were −0.060 and −0.013, excluding 0, and the mediation effect was −0.033. Thus, H2 was validated. When work–family conflict was used as the single mediating variable, the lower and upper BC confidence intervals of professionalization on job burnout through work–family conflict were −0.047 and −0.004, respectively, not including 0. The mediating effect was −0.021, so H4 was confirmed. When workload and work–family conflict were used as mediating variables together, the lower and upper BC confidence intervals for the specific indirect effects were −0.030 and −0.006, exclusive of 0, respectively, indicating that workload and work–family conflict could play a two-fold mediating role between professional level and job burnout, with a total mediating effect of −0.015, as verified by H5. Similarly, when job insecurity and work–family conflict, and job insecurity and workload were mediating variables at the same time, the lower and upper BC confidence intervals for the specific indirect effects were −0.042 and −0.008, −0.056 and −0.013, neither of which included 0. The total mediating effects were 0.022 and 0.032, indicating that job insecurity and work–family conflict, and job insecurity and workload were each able to act as a double between the professional level and job burnout mediating effect, which was verified for H6 and H7.

## 6. Discussion

### 6.1. Summary of Findings

Although previous studies have identified a range of antecedents that influence job burnout among construction workers, such as job stress, workload, and work–family conflict, there is a lack of exploration for the impact of professionalization on job burnout [78,85]. Based on the background of the Chinese construction industry, this paper developed a theoretical model to analyze whether professionalization contributes to construction workers’ job burnout and validated this model through SEM. It paid particular attention to the important mediating role of workload, job insecurity, and work–family conflict. This study provides practical evidence of the importance of professionalization in improving job burnout among construction workers, extending the scope of research on the antecedents of job burnout. Moreover, this study also fills the research gap as a theoretical system since the correction of job burnout is scarce in the construction industry from the perspective of professionalization. The main conclusions drawn from this study are as follows.

As expected, professionalization showed a high level of influence on job burnout with a more significant direct negative effect relationship. By promoting professionalization, construction workers’ job burnout can be directly and effectively alleviated. Furthermore, workload and work–family conflict were found to mediate the relationship between professionalization and job burnout, not only separately, but also together as continuous mediators. These results were justified on the basis of the COR theory as well as supporting and extending the conclusions made in previous studies. According to the COR theory, professionalization can enhance the employment quality and complement the resources of workers, specifically in terms of improved job characteristics. For example, the transformation and upgrading of the construction industry together with changes in production methods can reduce the labor intensity and mobility of construction workers. Moreover, by strengthening support in education, housing, and social security, it can help construction workers and their families to facilitate into urban life [24,56]. The reduction in workload and work–family conflict allows workers to have more resources for effective emotional and behavioral adjustment to prevent negative occupational psychology such as exhaustion and burnout.

Although job insecurity caused by low professionalization level did not directly cause job burnout in this study, it could exacerbate job burnout through workload and work–family conflict. This finding was consistent with the conclusions of Mostert [68] and Xanthopoulou et al. [75]. To ease their anxiety, workers often work harder to justify their worth to managers. In addition, bringing negative feelings resulting from insecurity into their families can exacerbate the conflict between work and family for workers. All of the above can worsen their job burnout.

### 6.2. Theoretical Implications

Job burnout has received much attention in previous studies considering that it can negatively affect project performance as well as causing workers’ unsafe behavior [23,91]. Studies on the antecedent variables of job burnout have confirmed the influence of single or several variables such as work–family conflict, work stress, and workload on it [5,22,56,85]. However, few studies have yet systematically examined whether and how professionalization can alleviate job burnout in the field of construction project management, including introducing workload, job insecurity, and work–family conflict as mediating variables.

Considering the special group characteristics of Chinese construction workers, this study creatively constructed a theoretical model based on the COR theory, with workload, job insecurity, and work–family conflict as mediating variables. The results revealed that an increase in professionalization level could directly ease the job burnout of construction workers. This study extends the existing body of knowledge on the precursors of job burnout, achieving an innovation in a research perspective.

This study also explored whether and how workload, job insecurity, and work–family conflict impact the relationship between professionalization and job burnout. The results supported the findings of Sun [5] and Liu et al. [85] in that workload and work–family conflict could impact the relationship between professionalization and job burnout. This study also captured the interaction process between professionalization, workload, work–family conflict, and job burnout, and found that a reduction in workload resulting from an increased professionalization level was the most effective way to alleviate burnout. In addition, job insecurity caused by a low professionalization level could reinforce job burnout through workload and work–family conflict in this study. Therefore, this factor cannot be ignored when discussing the impact of professionalization on job burnout. Although job burnout has been emphasized as an important topic in previous studies, most studies have only looked at the impact of one or a few variables of job burnout separately [24,56]. However, by establishing a theoretical model in which workload, job insecurity, and work–family conflict are identified as mediating variables, this study systematically explored how these three elements interact with each other to influence the interaction between professionalization and burnout. In addition, the importance of job insecurity in this study indicated that workers’ mental health deserves more attention in the professional development of the construction industry. This finding enhances the body of knowledge on the causes of burnout.

Finally, this study adapted the mature measurement scales to fit the background of the Chinese engineering construction field, improved the measurement scale of professionalization, and verified the practicality of it, further consolidating the theoretical basis of professionalization.

### 6.3. Practical Implications

As a psychological phenomenon, job burnout has a significant impact on the efficiency of project teams and the performance of projects and organizations [5]. However, the large scale, complexity, and integrated nature of construction projects requires workers to be under great pressure [92]. As a result, construction workers generally have a high risk of experiencing burnout [1]. This paper confirmed that construction companies could directly and effectively rectify construction workers’ job burnout by enhancing their professionalization, which provides a new direction to solve the problem of workers’ job burnout. This offers a fresh approach to dealing with job burnout in China and in many other nations and locations where construction workers are undergoing the industrialization process.

Furthermore, in the process of professionalization, construction companies should pay more attention to improving construction workers’ burnout by reducing workload and work–family conflict. To achieve this goal, a professional training system should be established oriented towards career development, as well as professional skills and safety knowledge training. On the one hand, this will help to transform construction workers into industrial workers. On the other hand, it can reduce the absolute working hours by means of increasing the working ability and efficiency of workers. At the same time, reasonable scheduling and work planning can also ensure that construction workers have sufficient time for rest and recovery, reducing exhaustion and burnout caused by long working hours, high labor intensity, and heavy work pressure. In addition, it is recommended to improve the leave system for workers. As construction workers work in the field all year round and find it difficult to balance work and family conflicts, companies could grant construction workers monthly leave to return home to attend to their family issues. While the above measures could help to reduce job burnout among construction workers, many construction firms find that they require a significant investment of time and material resources. In such circumstances, government and societal support are crucial. On the one hand, the improvement of the leave system needs to be aggressively encouraged by the government. On the other hand, specialized groups and societies can be established that can regularly plan vocational training for construction workers. This will decrease the number of resources used by businesses and raise the level of professionalism in training. Additionally, they could collaborate with construction companies to reach out to project sites and develop more specialized training for workers, taking into account the specifics of the project.

Finally, this study found that while job insecurity did not directly lead to job burnout, workers’ inner insecurities could exacerbate their emotional exhaustion through workload and work–family conflict. Therefore, attention should be paid to protecting the legal rights and interests of construction workers to lessen the indirect effects of job insecurity on job burnout. Government departments should raise the organizational level of employment, gradually replace the employment form of contractors or labor teams with small and micro-operating companies to guide workers to sign labor contracts, and establish stable employment relationships with enterprises. At the enterprise level, construction companies suggest reforming the year-end lump-sum wage settlement, clarifying the amount and timing of payment in conjunction with the content of labor contracts and paying wages in full each month as far as possible to enhance construction workers’ sense of security at work. These will contribute to raising the standard of living for construction workers, which will alleviate their burnout.

### 6.4. Limitations and Future Work

Many scholars have carried out research on job burnout in previous studies. However, few studies have systematically explored the mitigating effects of a high professional level on job burnout by developing a theoretical model. This study introduced three mediating variables (workload, job insecurity, and work–family conflict) and validated the model by SEM. This paper not only confirmed the findings of previous studies, but also presented new and meaningful conclusions that fill the gap in the field.

However, like all studies, this research had some limitations. Firstly, the sample data for this study was drawn from specific regions of China, with a total of 441 valid questionnaires. Although the requirements for sample size were met, considering the complexity of the model as well as the distribution and representativeness of the data, the range and number of sample sources could be expanded in future studies to evenly cover all project types, work types, and so on. At the same time, future research could further explore whether there are differences in the relationship of variables between workers in different job categories and ages. Secondly, the data obtained for this study in a specific time, space, and population were cross-sectional data where the constructs could only describe the relationship between variables within a short specific interval. However, the development of professionalization is a long-term process. Future research could analyze whether and how job burnout in construction workers changes within the same or multiple organizations over a relatively long period of time and compare it with the results of this study. In this way, the relationship between the variables could be further validated. Finally, although this study was completed anonymously and the workers were asked to respond based on their actual working conditions and personal feelings, the subjective nature of the questionnaire may have affected the authenticity of the results. Therefore, future research could incorporate methods such as semi-structured interviews and electroencephalography (EEG) experiments for further investigation.

## 7. Conclusions

Applying SEM, this study explored the influencing mechanism of professionalization on job burnout among Chinese construction workers, focusing on the mediating role of workload, job insecurity, and work–family conflict. The results showed that: (i) an increase in professionalization could be directly effective in alleviating job burnout among construction workers; (ii) workload and work–family conflict could play an independent and continuous mediating role between professionalization and job burnout; (iii) while job insecurity caused by a low professionalization level did not have a direct impact on job burnout, it could have an indirect impact on job burnout through workload and work–family conflict, respectively. From a theoretical aspect, this study innovatively conformed the impact of professionalization level on Chinese construction workers’ job burnout, contributing to our understanding the relationship between them. On the practical side, the pathway of the impact of professionalization level on job burnout provides directions for relevant government departments and construction companies to alleviate job burnout among Chinese construction workers, thereby advancing project success.

## Figures and Tables

**Table 1:** Demographic information of respondents (N = 441).

Variable	Categories	Number of Cases	Frequency (%)
Gender	Male	392	88.9
	Female	49	11.1
Age	Between 16 and 20	7	1.6
	Between 21 and 30	113	25.6
	Between 31 and 42	196	44.4
	Between 43 and 50	66	15.0
	Between 51 and 60	57	12.9
	Above 60	2	0.5
Marital status	Unmarried	103	23.4
	Married	338	76.6
Educational level	Primary school and below	18	4.1
	Junior high school	111	25.2
	Certificate or associate’s degree	58	13.1
	Senior high school	50	11.3
	Junior college	104	23.6
	Undergraduate and above	100	22.7
Project type	Construction project	213	48.3
	Municipal project	81	18.4
	Railway project	34	7.7
	Highway project	17	3.8
	Water conservancy and hydropower project	58	13.2
	Mining project	1	0.2
	Others	37	8.4
Seniority	Less than 5 years	144	32.7
	Between 6 and 10 years	128	29.0
	Between 11 and 15 years	98	22.2
	Between 16 and 20 years	27	6.1
	Greater than 20 years	44	10.0
Daily working hours	Less than 8 h	69	15.7
	Between 8 and 10 h	322	73.0
	Greater than 10 h	50	11.3
Average monthly income	Less than CYN 5000	76	17.2
	CYN 5001–8000	205	46.5
	CYN 8001–11,000	123	27.9
	CYN 11001–14,000	20	4.5
	CYN 14,000 and above	17	3.9

**Table 2:** Results of model fit indices for measurement model. (N = 441).

Model Fit Indices	Recommended Values	Values
Absolute fit measures		
χ^2^/df	≤2 ^a^; ≤5 ^b^	3.199
RMR	<0.1	0.084
GFI	≥0.9 ^a^; ≥0.8 ^b^	0.833
RMSEA	<0.08 ^a^; <0.1 ^b^	0.071
Incremental fit measures		
NFI	≥0.9 ^a^; ≥0.8 ^b^	0.908
IFI	≥0.9	0.935
TLI/NNFI	≥0.9	0.927
AGFI	≥0.9 ^a^; ≥0.8 ^b^	0.800
CFI	≥0.9	0.935
Parsimonious fit measures		
PGFI	≥0.5	0.697
PNFI	≥0.5	0.927
PCFI	≥0.5	0.071

Note: ^a^ equals acceptable; ^b^ equals marginal.

**Table 3:** Results of reliability and convergent validity for each construct. (N = 441).

Variable and Construct		Item	FL	CR	AVE	Cronbach’s Alpha
PL	PA	PA1	0.895	0.971	0.971	0.829
		PA2	0.949			
		PA3	0.949			
		PA4	0.918			
		PA5	0.885			
		PA6	0.875			
		PA7	0.898			
	JC	JC1	0.854	0.957	0.951	0.847
		JC2	0.953			
		JC3	0.950			
		JC4	0.920			
WL		WL1	0.672	0.819	0.603	0.808
		WL2	0.849			
		WL3	0.798			
JI		JI1	0.704	0.898	0.692	0.898
		JI2	0.749			
		JI3	0.944			
		JI4	0.905			
WFC		WFC1	0.802	0.878	0.706	0.907
		WFC2	0.829			
		WFC3	0.887			
		WFC4	0.855			
JB	EE	EE1	0.736	0.851	0.846	0.657
		EE2	0.842			
		EE3	0.849			
	CY	CY1	0.864	0.934	0.931	0.826
		CY2	0.923			
		CY3	0.937			
	LPE	LPE1	0.912	0.856	0.880	0.669
		LPE2	0.801			
		LPE3	0.726			

Note: PL = professionalization; PA = personal attributes; JC = job characteristics; WL = workload; JI = job insecurity; WFC = work–family conflict; JB = job burnout; EE = emotional exhaustion; CY = cynicism; LPE = low professional efficacy; FL = factor loading; CR = composite reliability; AVE = average variance extracted.

**Table 4:** Descriptive statistics and correlation analysis. (N = 441).

Variable	Mean	SD	PA	JC	WL	JI	WFC	EE	CY	LPE
PA	4.163	0.874	**0.910**							
JC	3.871	1.042	0.693 **	**0.920**						
WL	2.834	0.856	−0.217 **	−0.341 **	**0.777**					
JI	2.598	1.039	−0.312 **	−0.410 **	0.605 **	**0.832**				
WFC	2.765	1.116	−0.325 **	−0.439 **	0.639 **	0.579 **	**0.840**			
EE	2.215	0.982	−0.488 **	−0.539 **	0.390 **	0.388 **	0.465 **	**0.811**		
CY	2.054	1.027	−0.517 **	−0.548 **	0.373 **	0.359 **	0.467 **	0.803 **	**0.909**	
LPE	1.842	0.906	−0.508 **	−0.476 **	0.316 **	0.372 **	0.387 **	0.653 **	0.768 **	**0.817**

Note: ** *p* < 0.01.

**Table 5:** Hypothesis testing results.

Hypothesis	Path	Path Coefficients	C.R.	*p*	Result
H1	PL→JB	−0.466	−7.507	***	Support
	PL→WL	−0.308	−6.559	***	
	WL→JB	0.198	2.313	*	
H2	PL→WL→JB	——	——	——	Support
	PL→JI	−0.494	−7.109	***	
	JI→JB	−0.025	−0.676	0.499	
H3	PL→JI→JB	——	——	——	Not support
	PL→WFC	−0.190	−3.305	***	
	WFC→JB	0.125	2.179	*	
H4	PL→WFC→JB	——	——	——	Support
	WL→WFC	0.681	7.297	***	
H5	PL→WL→WFC→JB	——	——	——	Support
	JI→WFC	0.166	3.794	***	
H6	PL→JI→WFC→JB	——	——	——	Support
	JI→WL	0.296	8.234	***	
H7	PL→JI→WL→JB	——	——	——	Support

Note: *** *p* < 0.001; * *p* < 0.05; C.R. = critical ratio.

**Table 6:** Results of regression analysis. (N = 441).

	Job Insecurity		Workload		Work–Family Conflict		Job Burnout	
	β	t	β	t	β	t	β	t
Gender	0.500	−1.964	−0.101	−0.910	0.054	0.428	−0.028	−0.271
Age	0.150	−0.231 *	0.053	1.085	−0.057	−1.030	−0.041	−0.887
Marital status	0.167	0.176	−0.022	0.221	−0.133	−1.215	−0.013	−0.138
Educational level	0.016	0.388	0.046	1.974 *	0.031	1.182	0.033	1.481
Seniority	0.056	0.889	−0.020	−0.603	0.035	0.938	−0.007	−0.216
Daily working hours	0.017	−0.803	0.013	1.994 *	0.012	0.158	0.134	2.139 *
Average monthly income	0.018	−0.707	0.007	0.178	0.024	0.562	−0.086	−2.431 *
Professionalization level	−0.493	−9.256 ***	−0.213	−4.972 ***	−0.153	−3.066 **	−0.434	−10.391 ***
Job insecurity			0.423	11.950 ***	0.323	7.000 ***	0.054	1.326
Workload					0.516	9.478 ***	0.155	3.125 **
Work–family conflict							0.140	3.495 ***
R^2^	0.199		0.403		0.509		0.692	
F	13.408 ***		32.260 ***		44.083 ***		35.897 ***	

Note: *** *p* < 0.001; ** *p* < 0.01; * *p* < 0.05.

**Table 7:** Analysis of mediation effect.

Path	Effect	Bootstrapping Bias-Corrected 95% CI		Result
		Lower	Upper	
Direct effect	−0.434	−0.516	−0.352	—
Indirect effect	−0.162	−0.221	−0.094	—
PL→WL→JB	−0.033	−0.060	−0.013	Supported
PL→WFC→JB	−0.021	−0.047	−0.004	Supported
PL→WL→WFC→JB	−0.015	−0.030	−0.006	Supported
PL→JI→WFC→JB	−0.022	−0.042	−0.008	Supported
PL→JI→WL→JB	−0.032	−0.056	−0.013	Supported

Note: CI = confidence interval.

## Appendix A

**Table A1:** Questionnaire items in measurement scales.

Construct			Code	Measurement Item
Professional level (PL)		Personal attributes (PA)	PA1	I have specific vocational skills or expertise.
			PA2	I can perform my work competently.
			PA3	I have a clear understanding of my position and responsibilities.
			PA4	I have a sense of responsibility and mission in my work.
			PA5	I dress and speak in a manner that meets the requirements of the company.
			PA6	I can comply with the company’s rules and regulations.
			PA7	I can protect the company’s reputation, image, and interests.
		Job characteristics (JC)	JC1	I am satisfied with the working and living environment on site.
			JC2	My wages and benefits are always fulfilled.
			JC3	My labor rights and interests are protected in my work group or company.
			JC4	I can regularly participate in training activities organized by the company or industry.
Workload (WL)			WL1	My work requires much physical activity every day.
			WL2	I often feel much time pressure due to the rate or pace at which the tasks or task elements occurred.
			WL3	I feel a bit overwhelmed to complete my work tasks every day.
Job insecurity (JI)			JI1	I am constantly worried about the possibility of being fired.
			JI2	My work is insecure with the possibility of losing it.
			JI3	Working hard is the only way to keep me from getting fired.
			JI4	I have to do good work if I want to keep it.
Work–family conflict (WFC)			WFC1	The nature of work makes it difficult for me to handle family matters and responsibilities.
			WFC2	Working away from home for a long time forces me to miss some family activities.
			WFC3	I have no energy to spend time or contact with my family due to the hard work.
			WFC4	My experience at work does not help me much in dealing with family matters.
Job burnout (JB)	Emotional exhaustion (EE)		EE1	The intensity of my daily work makes me feel physically and mentally exhausted.
			EE2	Working all day is really stressful for me.
			EE3	I feel a bit overwhelmed at work.
	Cynicism (CY)		CY1	I have become less and less interested in my work.
			CY2	I have lost the passion I used to have for my work.
			CY3	I am not as enthusiastic about my work as I used to be.
	Low professional efficacy (LPE)		LPE1	I feel my work is not very meaningful.
			LPE2	I feel my work is not very productive.
			LPE3	I feel my work does not contribute much.
